# Clinical and Histopathological Profile of Adults with Celiac Disease at Diagnosis and on a Gluten-Free Diet: A Cross-Sectional Observational Study

**DOI:** 10.3390/clinpract16050088

**Published:** 2026-04-30

**Authors:** Elena Maria Domsa, Ioana Para, Adela Viviana Sitar Taut, Teodora Atena Pop, Elena Ofelia Mosteanu, Laura Elena Gligor, Mihaela Elvira Cîmpianu, Antonia Gabriela Nitescu, Viorel Lucian Marina, Bogdan Nicolae Mucea, Vasile Andreica

**Affiliations:** 1 Department of Nursing, “1 Decembrie 1918” University of Alba Iulia, 510009 Alba Iulia, Romania; maria.domsa@uab.ro (E.M.D.); laura.gligor@uab.ro (L.E.G.); antonia.nitescu@uab.ro (A.G.N.); 2 4th Department of Internal Medicine, “Iuliu Hațieganu” University of Medicine and Pharmacy, 400006 Cluj-Napoca, Romania; ioana.para@yahoo.com (I.P.); adelasitar@yahoo.com (A.V.S.T.); teodorapop@gmail.com (T.A.P.); ofeliamosteanu@gmail.com (E.O.M.); andreicav@umfcluj.ro (V.A.); 3 Department of Social Sciences, “1 Decembrie 1918” University of Alba Iulia, 510009 Alba Iulia, Romania; lucian.marina@uab.ro (V.L.M.); bogdan.mucea@uab.ro (B.N.M.)

**Keywords:** celiac disease, gluten-free diet, intestinal manifestations, extraintestinal manifestations, associated diseases

## Abstract

**Background/Objectives:** Celiac disease (CD) is a chronic immune-mediated enteropathy triggered by gluten ingestion in genetically predisposed individuals, with a highly heterogeneous clinical presentation encompassing both intestinal and extraintestinal manifestations. Despite growing clinical awareness, diagnostic delay remains a significant challenge, and the evolution of clinical and histopathological features following gluten-free diet (GFD) initiation remains incompletely characterized. This study aimed to compare the clinical, serological, and histopathological profile of adults with newly diagnosed CD and those maintained on a long-term GFD. **Methods:** This prospective observational study enrolled 50 adult patients with biopsy-confirmed CD: 16 at the time of diagnosis (gluten-consuming) and 34 on a GFD for at least one year. All participants underwent standardized clinical assessment, serological testing (IgA anti-tissue transglutaminase [tTG] and/or IgA anti-endomysial antibodies [EMAs]), upper digestive endoscopy with duodenal biopsies graded according to the Marsh–Oberhuber classification, and evaluation for *Helicobacter pylori* co-infection. Between-group comparisons were performed using the Mann–Whitney U test, Fisher’s exact test, and chi-square test, as appropriate. **Results:** Newly diagnosed patients exhibited a significantly longer median duration from symptom onset to diagnosis compared to the GFD group (*p* = 0.03). Histopathological severity was greater in the newly diagnosed group, with more advanced Marsh–Oberhuber grades (*p* = 0.03). Among individual symptoms, bloating was significantly more frequent in newly diagnosed patients (*p* = 0.02). Notably, thrombocytosis was identified significantly more often in the newly diagnosed group compared to GFD patients (*p* = 0.02), representing a potentially underrecognized extraintestinal marker of active CD. Overall rates of intestinal and extraintestinal manifestations and CD-specific seropositivity did not differ significantly between groups. **Conclusions:** The clinical and histopathological profile of adults with celiac disease differs meaningfully between the time of diagnosis and during GFD adherence. Bloating and thrombocytosis were significantly more prevalent at diagnosis, with thrombocytosis emerging as a potentially underrecognized marker of active disease. Conversely, several manifestations persisted despite dietary treatment, underscoring the heterogeneous nature of CD across its clinical course. These findings may support earlier disease recognition and more individualized follow-up strategies in routine clinical practice.

## 1. Introduction

Celiac disease (CD) is a chronic, immune-mediated disorder triggered by the ingestion of gluten in genetically predisposed individuals and characterized by inflammation and villous atrophy of the small intestinal mucosa [1,2,3]. Once considered a rare pediatric condition, celiac disease is now recognized as a common, lifelong, systemic disorder that may present at any age and affect multiple organ systems [3,4,5]. Over the past decades, its reported prevalence has increased worldwide, reflecting improved diagnostic awareness, wider use of serological screening, and possibly a true increase in incidence [5,6].

Despite these advances, celiac disease remains substantially underdiagnosed, particularly in adults. Diagnostic delay is frequent and is often related to the heterogeneous clinical presentation of the disease [7,8,9]. Epidemiological studies consistently report a higher prevalence among women and an increased risk in first- and second-degree relatives of affected patients, as well as in individuals with other autoimmune disorders [5,7,10,11]. These data emphasize the need for heightened clinical suspicion, especially outside classical gastrointestinal presentations.

The clinical spectrum of celiac disease has evolved considerably in recent years. While classical symptoms such as chronic diarrhea, abdominal pain, bloating, and weight loss are still encountered, many patients present with non-classical or predominantly extraintestinal manifestations [12]. These include iron-deficiency anemia, metabolic bone disease, neurological symptoms, reproductive disorders, dermatological conditions, and psychiatric manifestations, which may precede or even replace gastrointestinal symptoms [13,14,15,16,17,18,19,20]. Consequently, an increasing proportion of patients are diagnosed with atypical or asymptomatic forms of the disease, further complicating timely recognition [5,12].

The diagnosis of celiac disease is based on a combination of clinical assessment, serological testing, histopathological evaluation of duodenal biopsies, and, in selected cases, genetic testing for HLA-DQ2 and HLA-DQ8 haplotypes [1,2,21,22]. However, seronegative celiac disease represents a particular diagnostic challenge, as patients may lack circulating disease-specific antibodies despite histological features suggestive of celiac disease [23,24,25,26,27]. In such situations, histopathology and genetic testing become essential tools for confirming the diagnosis [28,29,30,31,32].

Lifelong adherence to a gluten-free diet (GFD) remains the cornerstone of treatment and is generally associated with clinical improvement and mucosal recovery [1,2]. Nevertheless, several studies have shown that symptom resolution and histological healing are not universal, particularly in adults [33,34,35,36,37,38]. Persistent villous atrophy, ongoing symptoms, and extraintestinal manifestations have been reported despite dietary treatment, raising questions about adherence, duration of treatment, and the involvement of immune-mediated mechanisms beyond gluten exposure [13,20].

Comparative evaluations between newly diagnosed patients and those already following a gluten-free diet are therefore clinically relevant, as previous studies have documented significant differences in clinical presentation and symptom recovery between these two populations [9,12]. Such comparisons may provide insights into the natural course of the disease, identify features associated with delayed diagnosis, and highlight manifestations that may persist despite treatment [13,33,39]. 

However, real-life data addressing these differences remain limited, particularly in adult populations managed in routine clinical practice. In this context, distinguishing between features related to active disease at diagnosis and those persisting during follow-up on a gluten-free diet is of particular clinical importance. 

Therefore, the aim of this study was to describe and compare the clinical and paraclinical characteristics of patients with newly diagnosed celiac disease and those following a gluten-free diet, with particular emphasis on intestinal and extraintestinal manifestations, serological status, and histopathological findings.

## 2. Materials and Methods

### 2.1. Study Design and Participants

This analytical observational study included 50 adult patients diagnosed with celiac disease between 2015 and 2018. Patients were recruited from the "Prof. Dr. Octavian Fodor" Regional Institute of Gastroenterology and Hepatology and the 4th Medical Clinic of Cluj-Napoca.

Data were collected prospectively, while the analysis represents a cross-sectional comparison of two independent groups assessed at a single time point.

Participants were divided into two groups: patients with newly diagnosed celiac disease consuming a gluten-containing diet at the time of diagnosis (newly diagnosed CD group, *n* = 16) and patients with celiac disease following a gluten-free diet for at least one year (GFD group, *n* = 34). Adherence to the gluten-free diet was assessed based on patient self-report during clinical evaluation, including information regarding dietary compliance and possible gluten exposure. The larger proportion of patients in the GFD group reflects the distribution observed in routine clinical practice, where follow-up patients represent the majority of referrals to a tertiary gastroenterology center. The diagnosis of celiac disease was established according to contemporary international guidelines [1,2].

### 2.2. Ethical Approval

The study protocol was approved by the Ethics Committee of the "Iuliu Hațieganu" University of Medicine and Pharmacy, Cluj-Napoca, and by the Ethics Committee of the "Prof. Dr. Octavian Fodor" Regional Institute of Gastroenterology and Hepatology, Cluj-Napoca. All participants provided written informed consent prior to inclusion.

### 2.3. Clinical and Paraclinical Assessment

All patients underwent a comprehensive clinical evaluation based on a standardized study record sheet, which included demographic data, age at diagnosis, duration from symptom onset to diagnosis, dietary adherence, intestinal and extraintestinal manifestations, family history of celiac disease or other autoimmune conditions, and associated diseases.

Body mass index (BMI) was calculated and categorized as underweight (<18.5 kg/m^2^), normal weight (18.5–24.9 kg/m^2^), overweight (25–29.9 kg/m^2^), or obese (≥30 kg/m^2^). Adherence to the gluten-free diet was assessed by patient self-report and classified as strict or partial.

### 2.4. Serological and Laboratory Investigations

Serological testing included IgA anti-tissue transglutaminase (IgA tTG) antibodies measured by enzyme-linked immunosorbent assay and/or IgA anti-endomysial antibodies (IgA EMA) determined by indirect immunofluorescence. Values ≥ 10 U/mL for IgA tTG and ≥1:10 for IgA EMA were considered positive.

Hematological and biochemical parameters were evaluated to identify extraintestinal manifestations. Iron-deficiency anemia was diagnosed based on complete blood count and iron studies. Hypoalbuminemia was defined as serum albumin < 3.5 g/dL, hepatocytolysis as alanine and aspartate aminotransferase levels > 45 U/L, and vitamin deficiencies according to standard laboratory reference ranges.

### 2.5. Endoscopic and Histopathological Evaluation

All patients underwent upper gastrointestinal endoscopy with multiple duodenal biopsy sampling, including at least four specimens from the second portion of the duodenum and one to two samples from the duodenal bulb. Histopathological findings were classified according to the Marsh–Oberhuber classification as follows:

- Stage 0: absence of lymphocytic infiltrate, crypt hyperplasia and villous atrophy;
- Stage 1: presence of lymphocytic infiltrate, absence of crypt hyperplasia and villous atrophy;
- Stage 2: presence of lymphocytic infiltrate, crypt hyperplasia and absence of villous atrophy;
- Stage 3a: presence of lymphocytic infiltrate, crypt hyperplasia and partial villous atrophy;
- Stage 3b: presence of lymphocytic infiltrate, crypt hyperplasia and subtotal villous atrophy;
- Stage 3c: presence of lymphocytic infiltrate, crypt hyperplasia and total villous atrophy [40].

In patients with discordant serological and histological findings or in those already on a gluten-free diet at the time of testing, HLA-DQ2 and/or HLA-DQ8 genotyping was performed to support the diagnosis.

### 2.6. Helicobacter pylori Assessment

*Helicobacter pylori* infection was assessed using gastric biopsy samples in patients with endoscopic evidence of gastritis or peptic ulcer disease and by stool antigen or serological testing in the remaining patients.

### 2.7. Differential Diagnosis and Follow-Up

Differential diagnosis included exclusion of other causes of villous atrophy, such as infectious enteropathies, autoimmune enteropathy, inflammatory bowel disease, drug-induced enteropathy, immunodeficiency states, and non-celiac gluten sensitivity. Patients were evaluated either at the time of diagnosis or during routine follow-up visits, in accordance with disease management guidelines applicable at the time of the study [1,2].

### 2.8. Statistical Analysis

Statistical analyses were performed using MedCalc^®^ Statistical Software (version 19.6). Quantitative variables were tested for normality using the Shapiro–Wilk test and presented as means ± standard deviation or medians with interquartile ranges, as appropriate. Comparisons between groups were conducted using Student’s t-test for normally distributed variables and the Mann–Whitney U test for non-normally distributed variables.

Qualitative variables were expressed as frequencies and percentages and compared using the chi-square test or Fisher’s exact test. A *p* value < 0.05 was considered statistically significant. Due to the observational nature of the study and the specific patient population, a post hoc power analysis was conducted using G*Power (version 3.1.9.7) to estimate the study’s sensitivity. For the primary clinical and histopathological findings, the achieved statistical power (1–β) was found to range between 0.72 and 0.81, depending on the variables and the statistical tests applied (Chi-square or Fisher’s exact test). While we acknowledge that a larger sample size would further minimize the risk of Type II error (estimated at β = 0.19–0.28), these values indicate that the study was sufficiently sensitive to detect moderate-to-large effect sizes, providing a reasonable statistical basis for the observed differences between the groups.

## 3. Results

The study included 50 patients with celiac disease, of whom 16 were newly diagnosed and consuming a gluten-containing diet, and 34 were on a gluten-free diet for at least one year.

### 3.1. Demographic Characteristics

The demographic characteristics of the two groups are summarized in Table 1. Patients with newly diagnosed celiac disease had a mean age of 42.6 ± 12 years at study entry, while those on a gluten-free diet had a mean age of 44.6 ± 14.9 years. Female sex predominated in both groups (81.2% in newly diagnosed patients and 82.4% in the gluten-free diet group). Most patients originated from urban areas (75% and 79.4%, respectively). No statistically significant differences were observed between groups regarding age, sex, or background (*p* > 0.05).

### 3.2. Clinical and Paraclinical Characteristics

Clinical and paraclinical characteristics are presented in Table 2. Intestinal manifestations were reported by 43.8% of newly diagnosed patients and 50% of patients on a gluten-free diet, without a statistically significant difference between groups (*p* = 0.9). Similarly, extraintestinal manifestations were observed in 56.2% and 50% of patients, respectively (*p* = 0.9).

A family history of celiac disease was identified only in patients on a gluten-free diet (14.7%), while a family history of other autoimmune diseases was present in 18.8% of newly diagnosed patients and 14.7% of those on a gluten-free diet. These differences were not statistically significant (*p* > 0.05).

The mean age at diagnosis did not differ significantly between groups. However, the duration from symptom onset to diagnosis was significantly longer in newly diagnosed patients, with a median of 1 year (IQR 0–2), compared to 0 years (IQR 0–1) in the gluten-free diet group (*p* = 0.03). The lower median in the GFD group likely reflects a higher proportion of patients diagnosed rapidly through family or opportunistic screening, consistent with the shorter diagnostic delay reported in screened populations. In patients on a gluten-free diet, the median duration since diagnosis was 4 years (IQR 1–7), and the median duration of dietary treatment was 3 years (IQR 1–7). Based on patient self-report, adherence to the gluten-free diet was strict in 50% of cases and partial in the remaining 50%.

### 3.3. Nutritional Status and Serological Findings

Regarding body weight, 31.2% of newly diagnosed patients were underweight, 43.8% had normal weight, and 25% were overweight. In the gluten-free diet group, 23.5% were underweight, 58.8% had normal weight, 11.8% were overweight, and 5.9% were obese. No statistically significant differences in body mass index categories were observed between groups (*p* > 0.05).

IgA anti-tissue transglutaminase and/or IgA anti-endomysial antibodies were positive in 37.5% of newly diagnosed patients and 41.2% of patients on a gluten-free diet, with no significant difference between groups (*p* = 1). Notably, the persistence of seropositivity in a substantial proportion of patients on a gluten-free diet may reflect incomplete dietary adherence or ongoing mucosal inflammation despite treatment, and will be further discussed in the context of histopathological findings.

### 3.4. Histopathological Findings and Genetic Testing

Histopathological findings differed significantly between groups (*p* = 0.03) (Table 2). All newly diagnosed patients presented Marsh–Oberhuber grade 3 lesions, distributed as grade 3a in 25%, grade 3b in 37.5%, and grade 3c in 37.5% of cases. In contrast, patients on a gluten-free diet showed a wider distribution of histological grades, including Marsh–Oberhuber grades 0–2 in 41.2% of cases and grade 3 lesions in 58.8%, indicating partial but incomplete mucosal recovery in a substantial proportion of treated patients.

HLA-DQ2 and/or HLA-DQ8 genotyping was performed in patients with discordant serological and histological findings. Among those tested (*n* = 19), all were positive for HLA-DQ2 and/or HLA-DQ8.

### 3.5. Helicobacter pylori Infection and Complications

*Helicobacter pylori* infection was identified in 12.5% of newly diagnosed patients and 14.7% of patients on a gluten-free diet, without a statistically significant difference between groups (*p* > 0.05).

Complications, mainly infectious, were recorded in 12.5% of newly diagnosed patients and 17.6% of those on a gluten-free diet, also without statistically significant differences between groups (*p* > 0.05).

### 3.6. Intestinal Manifestations

The distribution of intestinal manifestations is shown in Table 3. In newly diagnosed patients, the most frequent symptoms were bloating (87.5%), abdominal pain (68.8%), and weight loss (68.8%). In the gluten-free diet group, abdominal pain and weight loss were most common (55.9% each), followed by diarrhea (50%) and bloating (50%).

Among all intestinal symptoms, bloating was significantly more frequent in newly diagnosed patients compared to those on a gluten-free diet (87.5% vs. 50%, *p* = 0.02), suggesting partial symptom resolution with dietary treatment. Notably, bloating persisted in half of the patients on a gluten-free diet, indicating that this symptom may not fully resolve despite treatment and warrants clinical attention during follow-up. No other intestinal manifestations differed significantly between groups (*p* > 0.05).

### 3.7. Extraintestinal Manifestations

Extraintestinal manifestations are detailed in Table 4. Fatigue/asthenia and peripheral neuropathy of the lower limbs were the most common findings in both groups. Thrombocytosis was significantly more prevalent in newly diagnosed patients (31.2%) compared to those on a gluten-free diet (5.9%) (*p* = 0.02), suggesting that this hematological abnormality represents an active-disease marker that responds to gluten withdrawal. No other extraintestinal manifestations showed statistically significant differences between groups (*p* > 0.05).

### 3.8. Associated Diseases

Associated conditions are summarized in Table 5. Disorders of the esophagus and stomach were the most frequently reported comorbidities in both groups (68.8% in newly diagnosed patients and 38.2% in the gluten-free diet group). Lactose intolerance was identified in 18.8% of newly diagnosed patients and 20.6% of those on a gluten-free diet. Autoimmune thyroiditis was present in 18.8% and 8.8% of patients, respectively, and allergic conditions were reported in 18.8% and 29.4%. Although the prevalence of associated diseases differed numerically between groups, no statistically significant differences were identified for any of the comorbidities assessed (*p* > 0.05).

## 4. Discussion

Celiac disease represents a diagnostic challenge due to its marked clinical heterogeneity and the frequent predominance of non-classical manifestations. The present study compared the clinical and paraclinical characteristics of patients with newly diagnosed celiac disease and those following a gluten-free diet, highlighting differences that are relevant for both diagnosis and long-term follow-up in routine clinical practice. As anticipated by the heterogeneous clinical spectrum outlined in the Introduction, our findings confirm that both intestinal and extraintestinal manifestations remain frequent across different stages of the disease.

In seronegative cases, the diagnosis was supported by an integrated approach including compatible clinical features and histopathological findings, together with HLA-DQ2/DQ8 genotyping. Notably, genetic testing was performed in all seronegative patients and provided important diagnostic support, as the presence of these haplotypes strengthens the likelihood of celiac disease in the absence of positive serology.

Consistent with previous reports, celiac disease in our cohort predominantly affected middle-aged women, a finding in line with epidemiological data showing higher prevalence in females across different age groups [4,5,41,42,43]. Most patients originated from urban areas, similar to observations reported by Ramakrishna et al., although without statistically significant differences between urban and rural populations [6]. These demographic similarities between groups support the comparability of the studied populations.

One of the most important findings of this study was the significantly longer interval between symptom onset and diagnosis in newly diagnosed patients. Delayed diagnosis remains a well-recognized issue in celiac disease, particularly in adults presenting with mild or atypical symptoms. Previous studies reported median diagnostic delays ranging from 3 to over 5 years [7,8,9], whereas in our cohort, the delay was shorter, possibly reflecting increased awareness and improved diagnostic strategies in recent years. Regarding the duration of the gluten-free diet, Casellas et al. and Agarwal et al. described a median of 4 years, comparable to the findings in our study [44,45]. Adherence to the gluten-free diet varies widely in the literature, with strict compliance reported in 36% to 96% of patients [46,47]; in our cohort, strict adherence was reported in 50% of cases.

Regarding nutritional status, numerous studies have shown that celiac disease is not exclusively associated with underweight, and that normal-weight, overweight, and obese patients may also be affected. Statistical data indicate that 8.8–20.8% of patients are overweight and 0–6% are obese at the time of diagnosis [48,49,50,51]. In the study by Norsa et al., 11.4% of patients on a gluten-free diet were overweight and 8.8% were obese [49]. Our findings are broadly consistent with these reports, with overweight being observed more frequently among newly diagnosed patients compared to reference studies.

Histopathological evaluation revealed significant differences between groups (*p* = 0.03), with all newly diagnosed patients presenting advanced Marsh–Oberhuber grade 3 lesions, while a substantial proportion of patients on a gluten-free diet continued to exhibit persistent villous atrophy. Although mucosal healing is expected after dietary treatment, several studies have shown that complete recovery is not universal, especially in adults [33,34,35]. Similar to the findings of Haere et al., Lee et al., and Bardella et al., a considerable proportion of patients in our GFD group remained classified as Marsh grade 3 despite treatment, with rates of persistent villous atrophy reported at 49.5%, 79%, and 62%, respectively [36,37,38]. Partial adherence to the gluten-free diet, reported by half of the treated patients, may have contributed to this persistence. The lower severity of histopathological lesions observed in patients on a gluten-free diet is consistent with previous studies demonstrating mucosal healing following dietary treatment [52,53,54,55,56]. However, given the cross-sectional design of our study, no direct conclusions can be drawn regarding histological improvement over time.

An important observation was the high proportion of seronegative patients at study entry: 62.5% of newly diagnosed patients and 58.8% of those on a gluten-free diet had negative serology, with nine patients in the GFD group remaining seronegative since diagnosis. Seronegative celiac disease is a recognized but incompletely understood entity, with reported prevalence ranging between 1% and 28% [23,24].

The proportion observed in our cohort is therefore higher than that reported in most studies and may be explained by several methodological and biological factors. Selective IgA deficiency, a well-recognized cause of false-negative serological results, was not systematically assessed in all patients and may have contributed to the observed findings [57,58]. In addition, variability in serological assays, including differences in sensitivity and laboratory techniques, as well as the timing of testing in relation to gluten exposure, may have influenced antibody detection [59,60].

Previous studies have described the clinical profile of seronegative patients, including those by Bernardo et al. and Kotze et al., while Aziz et al. reported that 31% of patients with villous atrophy in a large prospective cohort had seronegative celiac disease [25,26,27]. In our cohort, the diagnosis was supported by histopathological findings and HLA-DQ2/DQ8 genotyping, with all tested patients being positive [1,2].

The mechanisms proposed for seronegativity include immune complex formation between anti-TG2 autoantibodies and tissue transglutaminase, preventing antibody circulation, as well as associated immunoglobulin deficiencies [28,29,30,31,32]. Despite careful exclusion of alternative causes of villous atrophy, seronegative enteropathies remain a diagnostic challenge, and some degree of diagnostic uncertainty cannot be entirely excluded [61]. Therefore, these findings should be interpreted with caution and may reflect study-specific factors.

Among intestinal manifestations, bloating was significantly more frequent in newly diagnosed patients compared to those on a gluten-free diet (87.5% vs. 50%, *p* = 0.02). This finding likely reflects active mucosal inflammation and malabsorption at diagnosis and is consistent with previous reports describing bloating as one of the most common gastrointestinal symptoms in untreated celiac disease [9,12,23,62]. Several studies have shown that abdominal bloating is frequently reported both at diagnosis and during follow-up, often overlapping with functional gastrointestinal symptoms [63,64].

Notably, bloating persisted in a substantial proportion of patients on a gluten-free diet, suggesting incomplete symptom resolution despite dietary treatment. This observation is in line with previous studies indicating that a significant subset of patients continue to experience gastrointestinal symptoms, including bloating, even after long-term adherence to a gluten-free diet [63,65]. Possible explanations include persistent low-grade inflammation, altered gut motility, microbiota changes, or the coexistence of functional gastrointestinal disorders. These findings highlight the multifactorial nature of symptom persistence in celiac disease and underscore the importance of continued clinical monitoring during follow-up.

Among extraintestinal manifestations, thrombocytosis was significantly more prevalent in newly diagnosed patients (31.2% vs. 5.9%, *p* = 0.02), a finding that may be related to systemic inflammation or reactive thrombocytosis secondary to iron deficiency, both frequently described in active celiac disease [13,14,15,16,17,18,19]. Previous studies have reported that hematological abnormalities, including thrombocytosis, may be present at diagnosis and tend to improve following the initiation of a gluten-free diet, reflecting disease activity [66,67].

The marked reduction in thrombocytosis prevalence observed in our cohort after dietary treatment is consistent with these findings and supports the hypothesis that this hematological abnormality may be associated with active disease. However, this observation should be interpreted with caution. Platelet counts were available as numerical values for all patients; however, for the purposes of the present analysis, thrombocytosis was evaluated as a categorical variable, which may limit a more detailed quantitative interpretation.

In the absence of clinical evidence suggesting a clonal hematologic disorder, the observed thrombocytosis is more likely reactive. Previous studies have shown that thrombocytosis in celiac disease may be related to chronic inflammation, iron deficiency, or other systemic effects associated with active disease [66,67]. In inflammatory enteropathies more broadly, iron deficiency has also been associated with secondary thrombocytosis and platelet activation [68,69]. Therefore, in our cohort, thrombocytosis may represent a nonspecific marker of active disease rather than a disease-specific feature. Further studies incorporating more detailed quantitative analysis of platelet levels may help clarify the clinical significance of this observation.

Extraintestinal manifestations were highly prevalent in both groups (56.2% newly diagnosed, 50% GFD), supporting the concept that celiac disease is a systemic disorder rather than a purely gastrointestinal condition. Peripheral neuropathy, fatigue, hematological abnormalities, and metabolic bone disease were among the most frequent findings, explicable by malabsorption and immune-mediated mechanisms involving transglutaminase 2, transglutaminase 3, transglutaminase 6, synapsin 1, gangliosides, and collagen autoantigens [15,20,70,71]. The persistence of certain manifestations despite dietary treatment, also reported by Jericho et al. and Sansotta et al., suggests delayed or incomplete clinical response in adults, particularly in females and in those with long-standing disease or suboptimal dietary adherence [13,20,39]. Moreover, ongoing research into microRNA expression profiles and novel nanoparticle-based diagnostic approaches may contribute to better characterization of extraintestinal disease mechanisms in the future [52,53].

The prevalence of *Helicobacter pylori* infection in our cohort (12.5% newly diagnosed, 14.7% GFD) was comparable to that reported by Mrabet et al. (14.5%) and Lasa et al. (12.5%), supporting the notion that the association between *Helicobacter pylori* and celiac disease remains controversial and incompletely elucidated [54,55,56]. The occurrence of infectious complications, including Clostridium difficile infection and pneumococcal disease, was consistent with earlier reports. In the study by Emilsson et al., infections were described in 19% of patients with healed mucosa and in 24% of those with persistent villous atrophy, suggesting that ongoing mucosal damage contributes to increased infection susceptibility [72,73].

Associated autoimmune and non-autoimmune conditions observed in our study, including autoimmune thyroiditis, psoriasis, hepatic disorders, and gastrointestinal comorbidities, are consistent with previous reports [9,12,23,74]. While some conditions, such as psoriasis and autoimmune liver diseases, have been described with increased frequency in patients with celiac disease [5,75], these associations were not the primary focus of the present study. The distribution of gastrointestinal comorbidities in our cohort was generally in line with the literature, without significant differences between newly diagnosed patients and those on a gluten-free diet [76,77,78]. 

Rare associations were also identified in our cohort, including isolated cases of Biermer anemia, autoimmune hemolytic anemia, and Alport syndrome, as well as two cases of common variable immunodeficiency. These findings are in line with the limited data available in the literature [79,80,81,82,83] and further highlight the complex and heterogeneous clinical spectrum of celiac disease. However, such associations were not a primary focus of the present study [3,84,85].

### 4.1. Clinical Implications

These findings emphasize the need for heightened clinical suspicion of celiac disease, even in the absence of classical gastrointestinal symptoms. Extraintestinal manifestations—particularly thrombocytosis and peripheral neuropathy—and hematological abnormalities may represent key diagnostic clues in patients with atypical presentations. Persistent histological damage and symptoms in treated patients underline the importance of long-term monitoring, dietary adherence assessment, and individualized follow-up strategies in adult celiac disease management.

### 4.2. Limitations

We acknowledge the relatively small sample size as a limitation of our study. However, the post hoc power analysis suggests that for the main significant outcomes—such as the Marsh–Oberhuber distribution, prevalence of bloating, and thrombocytosis—the study reached a power level close to or exceeding the 80% threshold. This suggests that the clinical differences observed are robust, although results regarding secondary variables should be interpreted with caution due to the higher potential for Type II error.

Although the data were collected prospectively, the present study design is cross-sectional, involving two independent cohorts assessed at a single time point. Therefore, no conclusions can be drawn regarding temporal relationships, disease progression, or response to a gluten-free diet. 

A major limitation of this study is the assessment of adherence to the gluten-free diet based solely on patient self-report. This approach is subject to recall bias, reporting bias, and unintentional gluten exposure, and may not accurately reflect true dietary compliance. No validated adherence questionnaires, structured dietitian assessments, or objective surrogate markers were systematically used.

This limitation is particularly relevant given that a considerable proportion of patients reported only partial adherence and that persistent Marsh 3 lesions were observed in some cases. Therefore, the interpretation of findings in the gluten-free diet group should be made with caution, as inaccuracies may influence the observed results in adherence assessment.

Future studies should incorporate validated tools for assessing dietary adherence, including standardized questionnaires, dietitian-led evaluations, or objective biomarkers. Such approaches would also allow for a more precise evaluation of the individual response to a gluten-free diet. 

## 5. Conclusions

Celiac disease presents with a wide spectrum of clinical and paraclinical manifestations, which may contribute to underdiagnosis in clinical practice. The findings of the present study suggest the heterogeneity and clinical polymorphism of the disease, including both intestinal and extraintestinal features, as well as associated conditions.

The observed differences between newly diagnosed patients and those on a gluten-free diet highlight potential patterns related to disease evolution and management. However, given the relatively small and uneven sample size, these findings should be interpreted with caution.

Therefore, the results of this study should be considered exploratory and hypothesis-generating. Further research on larger, multicenter cohorts is needed to validate these observations and to better define the clinical and paraclinical profile of patients with celiac disease.

## Figures and Tables

**Table 1:** General patient information.

Variable		Newly Diagnosed CD (*n* = 16)	CD on GFD (*n* = 34)	*p* Value
Age at study entry (mean age, years; ±S.D.)		42.6 ± 12	44.6 ± 14.9	0.6
Sex, *n* (%)	female	13 (81.2)	28 (82.4)	1
	male	3 (18.8)	6 (17.6)	
Background, *n* (%)	urban	12 (75)	27 (79.4)	1
	rural	4 (25)	7 (20.6)	

Abbreviations: *n*, number; S.D., standard deviation.

**Table 2:** Clinical and paraclinical characteristics of the patients studied.

Variable		Newly Diagnosed CD (*n* = 16)	CD on GFD (*n* = 34)	*p* Value
Intestinal manifestations, *n* (%)		7 (43.8)	17 (50)	0.9
Extraintestinal manifestations, *n* (%)		9 (56.2)	17 (50)	0.9
Family history, *n* (%)	celiac disease	-	5 (14.7)	0.1
	other autoimmune diseases	3 (18.8)	5 (14.7)	0.6
Age at diagnosis (mean age, years; ±S.D.)		42.6 ± 12	38.2 ± 17.8	0.3
Time between onset and diagnosis (median, years)		1 (0–2)	0 (0–1)	0.03
Time between diagnosis and entering into the study (median, years)		-	4 (1–7)	
Duration of gluten-free diet (median, years)		-	3 (1–7)	
GFD adherence, *n* (%)	strict	-	17 (50)	
	partial	-	17 (50)	
Body weight, *n* (%)	underweight	5 (31.2)	8 (23.5)	0.4
	normal weight	7 (43.8)	20 (58.8)	
	overweight	4 (25)	4 (11.8)	
	obesity	-	2 (5.9)	
IgA tTG and/or IgA EMA antibodies, *n* (%)	positive	6 (37.5)	14 (41.2)	1
	negative	10 (62.5)	20 (58.8)	
Pathological examination, *n* (%)	Marsh–Oberhuber 0	-	8 (23.5)	0.03
	Marsh–Oberhuber 1	-	2 (5.9)	
	Marsh–Oberhuber 2	-	4 (11.8)	
	Marsh–Oberhuber 3a	4 (25)	9 (26.5)	
	Marsh–Oberhuber 3b	6 (37.5)	8 (23.5)	
	Marsh–Oberhuber 3c	6 (37.5)	3 (8.8)	
HLA-DQ2 and/or HLA-DQ8, *n* (%)	positive	10 (100)	9 (100)	
*Helicobacter pylori*, *n* (%)	positive	2 (12.5)	5 (14.7)	1
	negative	14 (87.5)	29 (85.3)	
Complications, *n* (%)		2 (12.5)	6 (17.6)	1

Abbreviations: *n*, number; S.D., standard deviation; HLA, human leukocyte antigen.

**Table 3:** Intestinal manifestations in the patients studied.

Variable	Newly Diagnosed CD (*n* = 16)	CD on GFD (*n* = 34)	*p* Value
Bloating, *n* (%)	14 (87.5)	17 (50)	0.02
Nausea, *n* (%)	3 (18.8)	5 (14.7)	1
Vomiting, *n* (%)	3 (18.8)	7 (20.6)	1
Diarrhea, *n* (%)	7 (43.8)	17 (50)	0.9
Constipation, *n* (%)	6 (37.5)	8 (23.5)	0.3
Abdominal pain, *n* (%)	11 (68.8)	19 (55.9)	0.5
Loss of appetite, *n* (%)	-	5 (14.7)	0.1
Weight loss, *n* (%)	11 (68.8)	19 (55.9)	0.5

Abbreviations: *n*, number.

**Table 4:** Extraintestinal manifestations in the study patients.

Variable	Newly Diagnosed CD (*n* = 16)	CD on GFD (*n* = 34)	*p* Value
Fatigue/Asthenia, *n* (%)	12 (75)	27 (79.4)	0.7
Vitamin deficits, *n* (%)	5 (31.2)	6 (17.6)	0.2
Aphthous stomatitis, *n* (%)	2 (12.5)	5 (14.7)	1
Dermatitis herpetiformis, *n* (%)	2 (12.5)	-	0.09
Iron-deficiency anemia, *n* (%)	6 (37.5)	9 (26.5)	0.5
Thrombocytosis, *n* (%)	5 (31.2)	2 (5.9)	0.02
Thrombocytopenia, *n* (%)	2 (12.5)	1 (2.9)	0.2
Hemorrhages, *n* (%)	4 (25)	13 (38.2)	0.5
Amenorrhea, *n* (%)	1 (7.7)	4 (14.3)	1
Oligomenorrhea/Polymenorrhea, *n* (%)	-	3 (10.7)	0.5
Spontaneous abortion, *n* (%)	4 (30.8)	6 (21.4)	0.6
Headache, *n* (%)	1 (6.2)	10 (29.4)	0.08
Peripheral neuropathy of lower limbs, *n* (%)	10 (62.5)	19 (55.9)	0.8
Anxiety, *n* (%)	1 (6.2)	3 (8.8)	1
Depression, *n* (%)	-	2 (5.9)	1
Schizophrenia, *n* (%)	-	1 (2.9)	1
Hypoalbuminemia, *n* (%)	7 (43.8)	9 (26.5)	0.3
Hepatocytolysis syndrome, *n* (%)	4 (25)	8 (23.5)	1
Osteopenia/Osteoporosis, *n* (%)	5 (31.2)	13 (38.2)	0.8

Abbreviations: *n*, number.

**Table 5:** Associated diseases.

Variable	Newly Diagnosed CD (*n* = 16)	CD on GFD (*n* = 34)	*p* Value
Lactose intolerance, *n* (%)	3 (18.8)	7 (20.6)	1
Biermer anemia, *n* (%)	1 (6.2)	-	0.3
Autoimmune hemolytic anemia, *n* (%)	-	1 (2.9)	1
Type I diabetes mellitus, *n* (%)	-	1 (2.9)	1
Cancer, *n* (%)	2 (12.5)	1 (2.9)	0.2
Allergies, *n* (%)	3 (18.8)	10 (29.4)	1
Allergic rhinitis, *n* (%)	1 (6.2)	2 (5.9)	1
Bronchial asthma, *n* (%)	-	2 (5.9)	1
Sarcoidosis, *n* (%)	1 (6.2)	-	0.3
Raynaud syndrome, *n* (%)	1 (6.2)	1 (2.9)	0.5
Sjogren syndrome, *n* (%)	-	1 (2.9)	1
Psoriasis, *n* (%)	2 (12.5)	-	0.09
Systemic lupus erythematosus, *n* (%)	-	1 (2.9)	1
Autoimmune thyroiditis, *n* (%)	3 (18.8)	3 (8.8)	0.3
Addison disease, *n* (%)	1 (6.2)	-	0.3
Esophagus and stomach diseases (gastritis, esophagitis, hiatal hernia, gastric ulcers), *n* (%)	11 (68.8)	13 (38.2)	0.08
Autoimmune hepatitis, *n* (%)	2 (12.5)	-	0.09
Primary biliary cirrhosis, *n* (%)	1 (6.2)	1 (2.9)	0.5
Microscopic colitis, *n* (%)	1 (6.2)	-	0.3
Irritable bowel syndrome, *n* (%)	1 (6.2)	2 (5.9)	1
Variable common immune deficiency, *n* (%)	-	2 (5.9)	1
Alport syndrome, *n* (%)	-	1 (2.9)	1

Abbreviations: *n*, number.

## Data Availability

The original contributions presented in this study are included in the article. Further inquiries can be directed to the corresponding author.

## Abbreviations

The following abbreviations are used in this manuscript:

ACG	American College of Gastroenterology
ALT	Alanine aminotransferase
AST	Aspartate aminotransferase
BMI	Body mass index
BSG	British Society of Gastroenterology
CD	Celiac disease
CVID	Common variable immunodeficiency
ELISA	Enzyme-linked immunosorbent assay
EMA	Anti-endomysial antibodies
ESsCD	European Society for the Study of Coeliac Disease
GFD	Gluten-free diet
HLA	Human leukocyte antigen
IBS	Irritable bowel syndrome
IgA	Immunoglobulin A
IQR	Interquartile range
tTG	Tissue transglutaminase (IgA anti-tissue transglutaminase antibodies)
TG2	Tissue transglutaminase 2
TG3	Transglutaminase 3
TG6	Transglutaminase 6
